# Supplementary material for: Patterns of Intron Gain and Loss in Fungi
Source: PLoS Biol. 2004 Nov 30;2(12):e422. doi: 10.1371/journal.pbio.0020422 (PMC532390; doi:10.1371/journal.pbio.0020422)
Supplement: Table S1 — Also available at http://genes.mit.edu/NielsenEtAl/. (4.3 MB ZIP). [file pbio.0020422.st001.zip › NielsenEtAl/html/480.html]

AN0504.1.NCU03436.1.MG03911.1.FG01464.1


```
 CLUSTAL W (1.82) Multiple Sequence Alignments - Introns Inserted


Sequence 1: NCU03436.1	388 aa
Sequence 2: MG03911.1	421 aa
Sequence 3: FG01464.1	401 aa
Sequence 4: AN0504.1	393 aa
Alignment Length: 428 aa
Number Identitical Residues: 255 aa
Alignment Score (without introns) 11893


MG03911.1 	-MASTVPKPGPANLRPGAGLDEWLEEAKRCHYLPEAVMKQLCEMVKEVLMEE1SNIQPVV
NCU03436.1	--MSTIPKPGPANLRPGAGLDEWLEEAKQCHYLPEPVMKQLCEIVKEVLMEE1SNIQPVV
FG01464.1 	-MASSVPRPGPANLGPNAGLDEWLEEAKQCHYLPERVMKELCEKVKEILMEE1SNIQPVC
AN0504.1  	MTDSKVPQPGPAKLKRNAGPDEWLEAAKDCKYLSESHMKQLCEIVKEYMMEE1SNIQPVS
          	   *.:*:****:*  .** ***** ** *:**.*  **:*** *** :*** ****** 

MG03911.1 	TPVVICGDIHGQFYDLLELFRVAGGMPGETSVQAPQTATTVITSEDLEPPTEITDPKLRK
NCU03436.1	TPVTICGDIHGQFYDLLELFRVAGGMPGETNVQAPKTATTVITSEDIEPPTEITDPNLKK
FG01464.1 	TPVTVCGDIHGQFYDLLELFRVSGGMPGESNVQAPKTATTVITSDDIEPPTEITNPKLRK
AN0504.1  	TPVTVCGDIHGQFYDLLELFRVSGGMPDASLAEPPKTSSAVITSDDIEPPTTITDPELRK
          	***.:*****************:****. : .:.*:*:::****:*:**** **:*:*:*

MG03911.1 	RIRS------SSAGGATEEGGGDSADQPAQPADATSEKSTAAPSPAADTRFVFLGDFVDR
NCU03436.1	KIKS------STD---SEAGAGSEKES---PSEETPQSSANTGSQSADTRFVFLGDFVDR
FG01464.1 	KIKASGENATSSGTEEAEAGEGDEDPDATMADRLESGVTVNSTSQSADNRYIFLGDFVDR
AN0504.1  	KLGK---------PGTAGDDDDDDDDNNENAGQKEKSSSSGTSEIAVNRNFVFLGDYVDR
          	::              :  . ...  .   .       :  : . :.: .::****:***

MG03911.1 	GYFSLETFTLLMCLKAK2YPDRIVLVRGNHESRQITQVYGFYEECQQKYGNASVWKACCQ
NCU03436.1	GYFSLETFTLLMCLKAK2YPDRIVLVRGNHESRQITQVYGFYEECQQKYGNASVWKACCQ
FG01464.1 	GYFSLETFTLLMCLKAK2YPDRIVLVRGNHESRQITQVYGFYEECQMKYGNASVWKACCH
AN0504.1  	GYFSLETLTLLLCLKAK2YPDRVTLVRGNHESRQITQVYGFYEECLQKYGNASVWKACCQ
          	*******:***:***** ****:.*********************  ************:

MG03911.1 	VFDFLVLAAIVDGSVLCVHGGLSPEIRTIDQIRVVARAQEIPHEGAFCDLVWSDPEDIDT
NCU03436.1	VFDFLVLAAIVDGTVLCVHGGLSPEIRTIDQIRVVARAQEIPHEGAFCDLVWSDPEDVDT
FG01464.1 	VFDFLVLAAIIDGEILCVHGGLSPEIRTIDQIRVVARAQEIPHEGAFCDLVWSDPEDVET
AN0504.1  	VFDFMTLGAIIDGRVLCVHGGLSPEIRTLDQVRVVARAQEIPHEGAFCDLVWSDPDDVET
          	****:.*.**:** :*************:**:***********************:*::*

MG03911.1 	WAVSPRGAG~WLFGDKVATEFNHVNRLQTIARAHQLVNEGYK~VCSVLSAGLQVLSICFM
NCU03436.1	WAVSPRGAG~WLFGDKVATEFNHVNGLTTIARAHQLVNEGYK0-----------------
FG01464.1 	WAISPRGAG~WLFGDKVATEFNHVNGLKLIARAHQLVNEGYK0-----------------
AN0504.1  	WAVSPRGAG1WLFGDKVADEFCHVNDLTLIARAHQLVNEGYK~-----------------
          	**:****** ******** ** *** *  *************                  

MG03911.1 	PTLTLLAIQYHFPQKSVVTVWSAPNYCYRCGNVASIMAVDRDLNTKFSIFSAVPDEQRHV
NCU03436.1	---------FHFAEKSVVTVWSAPNYCYRCGNVASIMTVDRDQNTKFSIFSAVPDDQRHI
FG01464.1 	---------YHFPENSVVTVWSAPNYCYRCGNVASIMAVDKDLNPKFSIFSAVPDDQRHV
AN0504.1  	---------YHFSNNNVVTVWSAPNYCYRCGNLASVCEINEDLKPTFKLFSAVADDQRHV
          	         :**.::.****************:**:  ::.* :..*.:****.*:***:

MG03911.1 	PAGRRGPGDYFL
NCU03436.1	PAGRRGLGDYFL
FG01464.1 	PALKRGPGDYFL
AN0504.1  	PQSRGGRSEYFL
          	*  : * .:***
```
